# Oral Manifestations in Children With Neutropenia: A Systematic Review

**DOI:** 10.7759/cureus.89721

**Published:** 2025-08-10

**Authors:** Esther Kirubakaran, Sushmita Shan, Vivek K, Selvakumar Haridoss, Kavitha Swaminathan

**Affiliations:** 1 Pediatric and Preventive Dentistry, Sri Ramachandra Institute of Higher Education and Research, Chennai, IND

**Keywords:** case reports, children, cross-sectional studies, gingivitis, gsf refractory leukopenia, neutropenia, oral manifestations, periodontitis, systematic review, ulcers

## Abstract

Neutropenia is a hematological disorder marked by low neutrophil counts, predisposing affected children to severe infections, including those in the oral cavity. Early identification of oral signs may facilitate timely hematological referral and intervention. This systematic review investigated oral manifestations in neutropenic children and emphasized the diagnostic role of pediatric dentists. Following the Preferred Reporting Items for Systematic Reviews and Meta-Analyses) guidelines, five databases (PubMed, Scopus, EMBASE, Web of Science, and Ovid Medline) were searched up to April 2025, yielding 22 studies, including 19 case reports and three cross-sectional studies. The most frequently reported oral findings were chronic gingivitis, periodontitis, ulcers, and horizontal bone loss. Clinical presentation and severity varied among neutropenia subtypes. Due to heterogeneity, prevalence estimates were limited; however, one cross-sectional study reported gingival bleeding in 73.3% and mouth sores in 53.3% of neutropenic children. The review concluded that oral signs may serve as early indicators of neutropenia, but the current evidence is constrained by selection bias and inconsistent reporting. Pediatric dentists should interpret these manifestations as suggestive rather than diagnostic. Future studies should aim for standardized reporting and subtype-specific analysis to enhance the clinical value of oral indicators in neutropenia.

## Introduction and background

Neutrophils play a vital role in the innate immune response, particularly in defending against bacterial and fungal infections [1]. Neutropenia, defined as a reduced absolute neutrophil count (ANC), compromises this defense and predisposes affected individuals to recurrent infections. In children, neutropenia may be congenital, cyclic, autoimmune, or acquired due to infections or medications. The severity is typically categorized as mild (ANC: 1,000-1,500/µL), moderate (ANC: 500-1,000/µL), or severe (ANC: <500/µL), with classifications also based on bone marrow reserve and underlying etiology [2].

Neutrophils are the most abundant leukocytes in circulation and act as first-line defenders by migrating to infection sites, performing phagocytosis, and generating reactive oxygen species and antimicrobial peptides. In neutropenic individuals, reduced neutrophil counts impair microbial clearance, particularly in mucosal environments such as the oral cavity. Pediatric neutropenia often presents with systemic manifestations such as otitis media, respiratory tract infections, and gastroenteritis [3]. However, oral manifestations are frequently the first observable signs, including recurrent oral ulcers, desquamative gingivitis, periodontal breakdown, tooth mobility, and delayed healing [4,5]. Studies have shown that mutations in the *ELANE* gene, implicated in severe congenital and cyclic neutropenia, may exacerbate periodontal destruction [6,7]. Management with granulocyte colony-stimulating factor (G-CSF) therapy has shown promise in increasing ANC and reducing oral complications [8]. Along with systemic treatment, stringent oral hygiene, oral prophylaxis, and a preventive treatment plan help control the oral manifestations and improve the quality of life [9].

Pediatric dentists are often among the first clinicians to observe these oral manifestations during routine check-ups. While systemic features of neutropenia have been well-documented in hematology literature, there is limited consolidated evidence focusing on the spectrum of oral findings in affected children. Early recognition of oral signs by dental professionals can facilitate timely hematologic referral and reduce the risk of life-threatening systemic infections, including sepsis [1].

The current review includes children under 18 years of age, in accordance with the World Health Organization’s definition of the pediatric age group [10]. Therefore, this systematic review aims to evaluate and synthesize existing evidence on the oral manifestations observed in children diagnosed with neutropenia to guide early recognition, interdisciplinary referral, and appropriate dental management.

## Review

Methodology

This systematic review was conducted and reported in accordance with the Preferred Reporting Items for Systematic Reviews and Meta-Analyses (PRISMA) 2020 guidelines. The review protocol was registered with PROSPERO (International Prospective Register of Systematic Reviews) under the registration number CRD42024626730.

The research question was formulated using the PEO framework: Population (P): children aged <16 years; Exposure (E): neutropenia (all subtypes); Outcome (O): oral manifestations (ulcers, gingivitis, periodontitis, etc.). The focused question was: What are the oral manifestations observed in children under 16 years of age diagnosed with neutropenia?

Inclusion and Exclusion Criteria

Studies were included if they reported oral manifestations in children under 18 years of age with any type of neutropenia. Eligible study designs comprised case reports, case series, cross-sectional studies, and cohort studies. Only articles published in the English language were considered for inclusion.

Studies were excluded if they focused on participants aged 18 years or older were excluded. Exclusion criteria also encompassed in vitro studies, animal studies, and editorials. Additionally, review articles or conference abstracts lacking full data were excluded from consideration.

Information Sources and Search Strategy

A comprehensive literature search was conducted across five electronic databases, namely, PubMed, Scopus, EMBASE, Web of Science, and Ovid Medline, up to April 2025. No restrictions were applied to the start date of publication. In addition, manual hand-searching of the reference lists of all included articles was performed to identify any further eligible studies. The complete search strategies for all databases are provided in the Appendices.

Study Selection

All search results were imported into Rayyan QCRI (web-based platform) for de-duplication and screening. Two reviewers (EK and SS) independently screened titles and abstracts according to eligibility criteria. Full texts of potentially relevant articles were retrieved and reviewed. Disagreements were resolved by discussion or adjudication by a third reviewer (SH). Cohen’s kappa coefficient was calculated to assess inter-reviewer agreement during full-text screening (threshold ≥0.75 considered acceptable) [11].

Data Extraction

A standardized data extraction form was developed and piloted on three studies to ensure consistency. Two reviewers (EK and SS) independently extracted the following variables: first author, year of publication, study design, age and sex of patients, sample size, type of neutropenia, systemic and oral manifestations, and diagnostic and treatment approaches. Extracted data were recorded in Google Sheets and cross-verified for accuracy.

Risk of Bias Assessment

The Joanna Briggs Institute (JBI) checklist was used to assess methodological quality of case reports and case series [12]. The JBI tool evaluates eight domains, with studies rated as low, moderate, or high risk of bias based on total affirmative scores. The JBI checklist includes the following eight domains: (1) clear description of the patient’s demographic characteristics, (2) clear description of the patient’s history and presenting condition, (3) clear description of the clinical condition at presentation, (4) clear description of diagnostic tests and assessment methods, (5) clear description of intervention(s) or treatment procedure(s), (6) post-intervention clinical condition, (7) adverse events or unanticipated events, and (8) takeaway lessons. Each study was evaluated on these domains and rated as having low, moderate, or high risk of bias based on the number of affirmative responses. Studies were classified as having low risk of bias if they received 6 or more “Yes” responses, moderate risk if they had 4-5 “Yes” responses, and high risk if they scored 3 or fewer. These cut-off criteria are consistent with JBI methodological guidance and prior systematic reviews utilizing this tool.

For cross-sectional studies, a modified Newcastle-Ottawa Scale (NOS) was applied, assessing: (1) selection, (2) comparability, and (3) outcome. A score ≥7 indicated good quality, 4-6 indicated fair quality, and ≤3 indicated poor quality [13]. Risk of bias assessment was performed independently by two reviewers and cross-checked for consistency. Discrepancies were resolved through discussion. These thresholds align with widely accepted conventions reported in previous reviews assessing non-randomized studies.

Results

**Figure 1:**
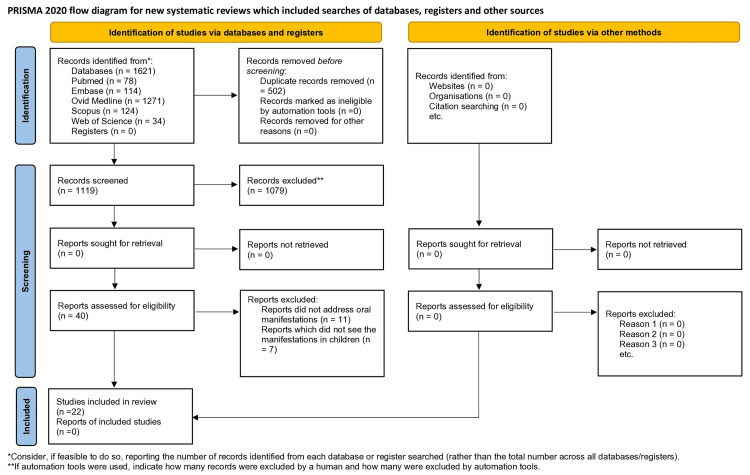
Preferred Reporting Items for Systematic Reviews and Meta-Analyses (PRISMA) 2020 flow diagram.

A total of 1,621 records were retrieved from electronic databases, including PubMed (n = 78), EMBASE (n = 114), Ovid Medline (n = 1,271), Scopus (n = 124), and Web of Science (n = 34). After removing 502 duplicates, 1,119 records remained for title and abstract screening. Of these, 1,079 articles were excluded based on predefined eligibility criteria. The full texts of 40 articles were assessed for eligibility. Ultimately, 22 studies met the inclusion criteria and were included in the systematic review. The reasons for exclusion of the remaining 18 articles (e.g., non-pediatric populations or lack of oral data) are detailed in the PRISMA flow diagram (Figure 1).

Study Characteristics

The 22 included studies (published between 1978 and 2025) comprised 19 case reports or case series and three cross-sectional studies. The characteristics of these studies, including country, study type, patient age, neutropenia subtype, and oral findings, are summarized in Table 1.

**Table 1:** Characteristics of the included studies. G-CSF: granulocyte colony-stimulating factor

Serial number	Author, year	Participant details	Type of neutropenia	Oral manifestations	Treatment options
Cross-sectional study					
1	Park et al. (2014) [14]	15 participants aged 6–16 years; 26 healthy controls	Idiopathic neutropenia	Oral ulcers, gingivitis, gingival bleeding, horizontal bone loss, early loss of tooth, pathological tooth mobility, and periapical infection	Antibiotic prophylaxis and endodontic procedure
2	Ye et al. (2011) [15]	4 patients aged 6–14 years	Severe congenital neutropenia	Two patients had periodontitis and gingivitis, while the other two were healthy	G-CSF and oral prophylaxis
3	Rezaei et al. (2007) [16]	17 patients aged 2–16 years	Severe congenital neutropenia	Oral ulcers, submandibular abscess, periodontitis, and dental abscess	Not mentioned
Case series					
4	Casey et al. (2011) [17]	A 6-year-old male and 4-year-old female	Benign familial neutropenia	Oral ulcers, reddish gingiva, stained teeth, periodontitis, and horizontal bone loss	Periodontal therapy and G-CSF
5	Mamishi et al. (2009) [18]	A 10-year-old male and a 9-month-old male	Severe congenital neutropenia	Oral ulcers and oral candidiasis	G-CSF and antibiotic prophylaxis
6	Carlsson et al. (2006) [19]	Two males aged 6 and 0.5 years and two females aged 7 and 14 years	Severe congenital neutropenia	Female patients: chronic gingivitis and alveolar bone loss. Male patient: (6-year-old): no periodontal manifestation. Male patient (0.5-year-old): chronic gingivitis	G-CSF and chlorhexidine mouthwash
7	Buduneli et al. (2006) [20]	11-year-old male and 5-year-old female	Familial neutropenia	Male patient: alveolar bone loss, localized periodontitis, and generalized gingivitis. Female patient: oral ulcers, alveolar bone loss, and gingivitis	Male patient: periodontal therapy. Female patient: oral prophylaxis, extraction of primary teeth, and space maintainer
8	Hakki et al. (2005) [21]	A 6-year-old male and a 3-year-old female	Severe congenital neutropenia	Male patient: severe periodontal and periapical infection, recurrent bacterial infection, tooth mobility, and gingivitis. Female patient: moderate gingival inflammation and tooth, and mobility	Male patient: oral prophylaxis, chlorhexidine mouthwash, and removable partial denture. Female patient: extraction, space maintainer, and oral prophylaxis
9	Kirstilä et al. (1993) [22]	Patient 1: 15-year-old male. Patient 2: 10-year-old female. Patient 3: 13-year-old male	Chronic neutropenia	Patients 1 and 2: severe periodontitis. Patient 1: generalized bone loss, tooth mobility. Patient 2: gingival hyperemia, plaque, gingival inflammation, incipient bone loss, and tooth mobility. Patient 3: oral ulcers	Patient 1: chlorhexidine mouthwash, anesthetic mouthrinse, and splint for mobility. Patient 2: periodontal maintenance. Patient 3: oral prophylaxis, chlorhexidine mouthrinse, and gingivectomy
10	Long et al. (1983) [23]	A 6-year-old male and 4-year-old female	Cyclic neutropenia	Male patient: halitosis, periodontal pocket, oral ulcers, severe gingivitis, tooth mobility, and alveolar bone loss. Female patient: deep periodontal pocket and horizontal bone loss	Oral prophylaxis
Case report					
11	Li et al. (2025) [24]	A 19-month-old male	Cyclic neutropenia	Oral ulcers, reddish lips, erythematous gingiva	G-CSF and antimicrobial therapy
12	Tao et al. (2024) [25]	A 3-year-old male	Severe congenital neutropenia	Severe gingivitis, periodontitis	G-CSF, oral prophylaxis, irrigation with saline and hydrogen peroxide, and oral antibiotics
13	Krasuska-Sławińska et al. (2023) [5]	A 2-year-old male	Severe congenital neutropenia	Chronic gingivitis, oral ulcers, early loss of tooth, tooth mobility, and periodontal pockets	G-CSF, antibiotic prophylaxis, oral prophylaxis, and photo-biomodulation
14	Dixon C et al. (2019) [4]	A 2-year-old female	Autoimmune neutropenia	Gingival hemorrhage, swollen gingiva, and horizontal bone loss	Chlorhexidine mouthwash and G-CSF
15	Chen et al. (2013) [9]	A 8-year-old male	Cyclic neutropenia	Oral ulcers, early loss of teeth, and severe alveolar bone	Oral antibiotics and G-CSF
16	Lu et al. (2012) [26]	A 6-year-old female	Cyclic neutropenia	Gingival recession, fiery red and soft gingiva, tooth mobility, deep dental caries, and recurrent oral ulcers	Oral prophylaxis, G-CSF, periodontal maintenance, root planing, and chlorhexidine mouthrinse
17	Zaromb et al. (2006) [27]	A 7-year-old male	Chronic benign neutropenia	Halitosis, premature loss of tooth, bleeding on probing, deep periodontal pocket, and aggressive periodontitis	Oral prophylaxis, antimicrobial mouthrinse, and G-CSF
18	Kamma et al. (1998) [28]	A 11-year-old female	Chronic idiopathic neutropenia	Premature loss of deciduous teeth, gingival bleeding, and tenderness	Professional prophylaxis, chlorhexidine mouthrinse, root planing, and antibiotic prophylaxis
19	Hastürk et al. (1995) [8]	A 6-year-old female	Chronic severe neutropenia	Severe tooth mobility, recurrent oral ulcers, alveolar bone loss, gingival inflammation, and enamel hypoplasia	G-CSF and oral prophylaxis
20	Baehni C et al. (1983) [29]	A 12-year-old male	Chronic neutropenia	Severe gingival inflammation, alveolar bone loss, and premature tooth loss	Not mentioned
21	Kalfwarf et al. (1981) [30]	A 3.5-year-old female	Chronic idiopathic neutropenia	Swollen erythematous gingiva, gingival recession, and alveolar bone loss	Oral prophylaxis
22	Reichart et al. (1978) [7]	A 7-year-old male	Chronic benign neutropenia	Gingivitis, periodontitis, advanced alveolar bone loss, and tooth mobility	Not mentioned

These studies, drawn from 12 countries, involved 68 pediatric patients aged between nine months and 16 years. Severe congenital neutropenia was the most frequently reported subtype (11 studies), followed by cyclic neutropenia (six studies), chronic idiopathic or familial forms (three studies), and autoimmune neutropenia (two studies). Oral manifestations included gingival inflammation (18 studies), periodontitis (16), recurrent oral ulcers (14), spontaneous gingival bleeding (12), alveolar bone loss (10), and tooth mobility or early exfoliation (eight). Cross-sectional studies reported broader spectra of disease [14-16], while case reports highlighted more severe and early-onset presentations [4,5,8,9,17-30].

Notably, the *ELANE* gene mutation, linked with severe congenital neutropenia (SCN) and cyclic neutropenia (CN), was associated with early-onset aggressive periodontitis and horizontal bone loss in five studies [17,19,20,22,23]. The cross-sectional study revealed that 11 out of 15 neutropenic children presented with gingival bleeding and eight with mouth sores, compared to age-matched controls [14].

While the case reports provided rich clinical detail, their findings may overrepresent severe presentations due to publication bias. Conversely, cross-sectional studies tended to report more variable presentations, possibly reflecting broader clinical spectra.

Treatment modalities reported across studies included G-CSF therapy, antibiotic prophylaxis, oral prophylaxis, chlorhexidine rinses, and, in severe cases, periodontal surgery or extraction with space maintenance.

Risk of Bias Assessment

**Figure 2:**
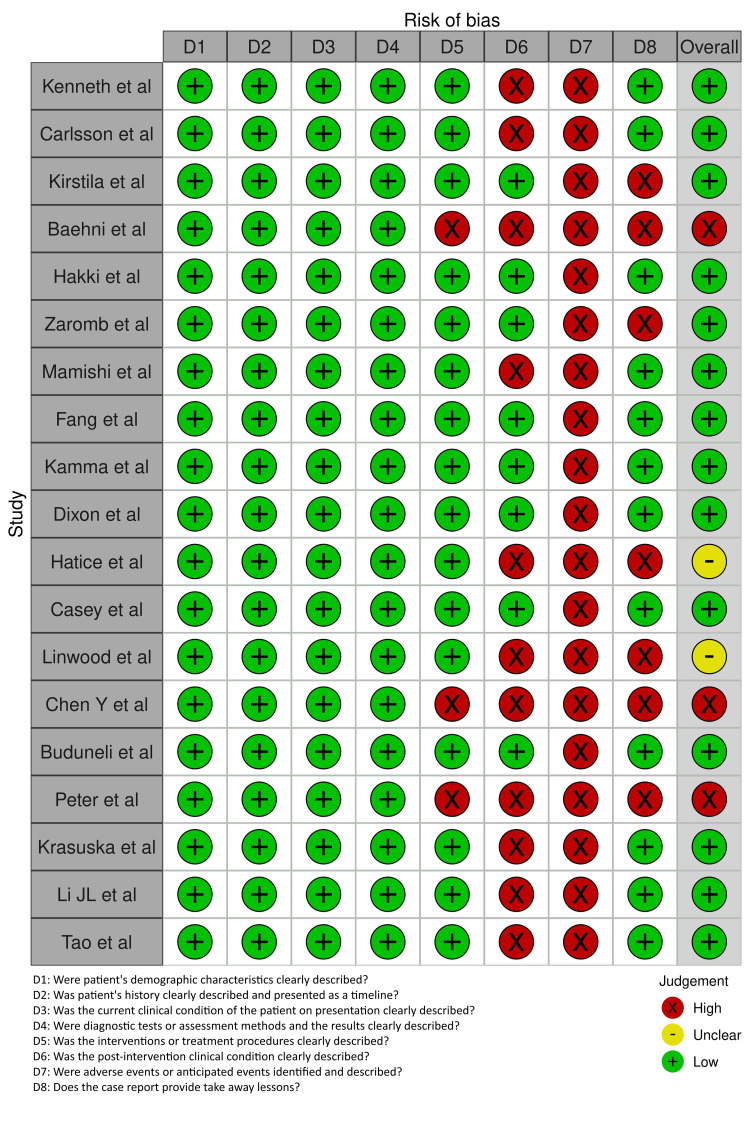
Risk of bias (Joanna Briggs Institute: case reports and series).

The methodological quality of the 19 case reports and case series was assessed using the JBI Critical Appraisal Checklist. Based on the overall scores, 14 studies were judged to have a low risk of bias, two moderate, and three high risk of bias (Figure 2).

The most common methodological limitations among high-risk studies included insufficient diagnostic detail, lack of longitudinal follow-up, and incomplete reporting of treatment outcomes. These weaknesses potentially limit the reliability of outcome attribution in complex clinical cases.

**Figure 3:**
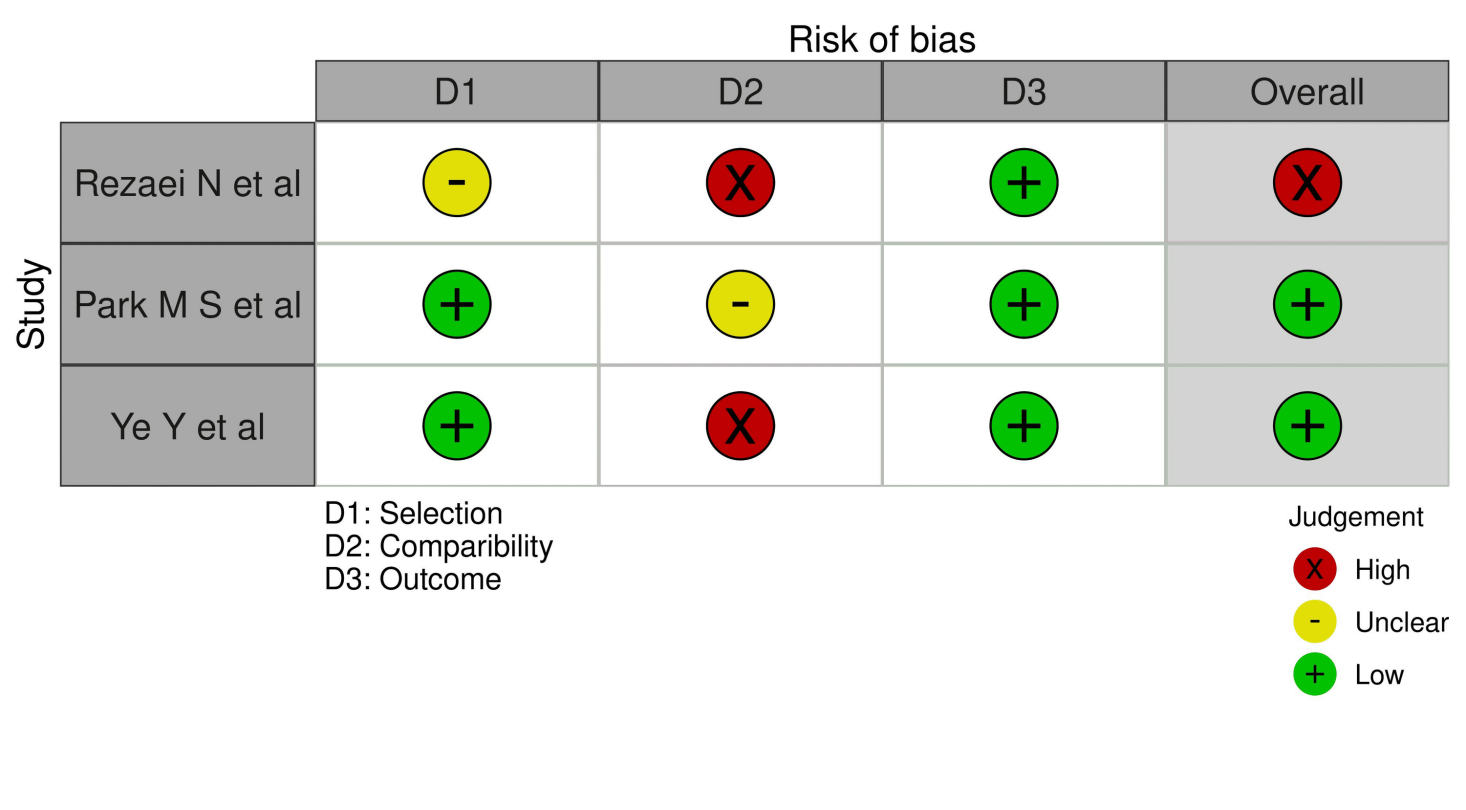
Risk of bias (Newcastle-Ottawa Scale: cross-sectional studies).

The three cross-sectional studies were evaluated using the modified NOS. Two studies achieved scores indicative of good methodological quality, particularly in the selection and outcome domains [14,15]. The third study was rated as fair quality, primarily due to limitations in sample comparability and control selection [16] (Figure 3).

Overall, the risk of bias across studies was moderate, with most case reports providing detailed clinical narratives but subject to selective reporting and publication bias. The cross-sectional studies, though fewer in number, contributed more structured comparative data, enhancing interpretive robustness.

Discussion

This systematic review synthesized evidence from 22 studies on the oral manifestations of neutropenia in children. The most frequently reported findings included gingival inflammation, periodontitis, recurrent ulcers, gingival bleeding, and early tooth loss. The clinical presentation and severity of these manifestations varied with the type and chronicity of neutropenia, as well as with patient age and genetic profile.

SCN and CN were associated with the most aggressive oral presentations, often beginning in early childhood. Multiple studies reported rapid periodontal breakdown and tooth mobility as early as two years of age in children with SCN, particularly those carrying mutations in the *ELANE* or *HAX1* genes [6,21,25].

The progressive loss of periodontal support, even with routine dental care, underscores the severity of immune dysregulation in these subtypes [5,19]. In CN, periodic depletion of neutrophils during 21-day cycles led to recurrent aphthous-like ulcers and gingival bleeding, most frequently affecting the buccal mucosa and tongue [31,32].

Autoimmune neutropenia, while generally self-limiting, also produced notable oral changes such as gingival hypertrophy, bleeding, and horizontal bone loss in children under three years [4]. However, these effects were typically less severe than those seen in congenital variants. In contrast, benign familial and chronic idiopathic neutropenia exhibited milder symptoms, with gingivitis and limited attachment loss being the most consistent findings [27,28]. These variations may reflect underlying differences in immune competency, oral hygiene practices, and microbial profiles [33].

Most of the included studies emphasized the role of G-CSF therapy in improving neutrophil counts and reducing oral lesion recurrence [8,20]. However, given the predominance of case-based literature, the clinical efficacy of G-CSF in mitigating long-term periodontal deterioration remains uncertain.

Moreover, few studies addressed the cost, access, or safety considerations associated with systemic therapy in pediatric populations. Adjunctive interventions such as chlorhexidine rinses, antibiotic prophylaxis, and professional oral hygiene were variably employed, with limited outcome standardization across studies.

Several methodological limitations were evident. The heterogeneity of study designs, ranging from single-patient case reports to small cross-sectional studies, precluded meaningful meta-analysis. The majority of studies lacked comparator groups, and oral findings were inconsistently reported or classified. Consequently, it remains difficult to determine whether the observed manifestations were directly attributable to neutropenia, as other contributing factors such as poor oral hygiene, coexisting systemic infections, nutritional deficiencies, or medication side effects could not be reliably excluded.

Additionally, the diagnostic criteria for both neutropenia and oral disease were not uniformly applied, limiting the ability to determine causal associations or prevalence estimates. The predominance of case reports introduces publication bias, as milder or asymptomatic cases may remain unreported. These factors collectively reduce the generalizability of the findings and highlight the need for standardized reporting guidelines in pediatric hematologic-dental research.

Despite these limitations, the review underscores an important clinical message: oral manifestations may serve as early indicators of underlying hematological disorders. Pediatric dentists are uniquely positioned to recognize atypical presentations such as unexplained gingival bleeding, recurrent ulcers, or premature tooth loss. Timely recognition and referral for hematologic evaluation could facilitate earlier diagnosis and management, potentially reducing systemic morbidity.

Future research should prioritize large-scale, multicenter studies using validated diagnostic and reporting frameworks to better define the spectrum and natural history of oral findings in pediatric neutropenia. Interdisciplinary collaboration between hematologists and pediatric dental professionals is essential to improve early detection, optimize care pathways, and enhance the quality of life of affected children.

## Conclusions

Children with neutropenia commonly present with gingival inflammation, periodontitis, recurrent ulcers, and early tooth loss. This review highlights that oral manifestations differ by neutropenia subtype: severe congenital and cyclic forms often lead to aggressive, early-onset periodontal disease, while autoimmune and idiopathic types tend to cause milder symptoms. Despite limitations due to case-based evidence, consistent findings across subtypes underscore the importance of early dental recognition. Pediatric dentists play a key role in identifying these signs and facilitating timely hematological referral. Future studies should focus on standardized, large-scale research to better define prevalence and clinical significance.

## Appendices

Search strategies by database

The search strategies used for different databases to identify relevant studies involved specific keywords and indexing terms.

PubMed (Medline)

(“oral manifestations”[MeSH Terms] OR (“oral”[All Fields] AND “manifestations”[All Fields]) OR “oral manifestations”[All Fields]) AND (“neutropenia”[MeSH Terms] OR “neutropenia”[All Fields] OR “neutropenias”[All Fields] OR “leukopenia”[All Fields]) AND (“child”[MeSH Terms] OR “child”[All Fields] OR “children”[All Fields])

EMBASE

‘oral manifestation’/exp OR ‘oral manifestation’:ti,ab AND (‘neutropenia’/exp OR neutropenia:ti,ab OR leukopenia:ti,ab) AND (‘child’/exp OR child*:ti,ab)

Scopus

TITLE-ABS-KEY(“oral manifestation”) AND TITLE-ABS-KEY(neutropenia OR leukopenia) AND TITLE-ABS-KEY(child OR children)

Web of Science

TS=(“oral manifestation”) AND TS=(neutropenia OR leukopenia) AND TS=(child OR children)

Ovid Medline

1. oral manifestations.mp. or Oral Manifestations/ 2. neutropenia.mp. or Neutropenia/ 3. leukopenia.mp. or Leukopenia/ 4. child.mp. or Child/ 5. 1 AND (2 OR 3) AND 4 6. Limit 5 to humans and English language
